# 

**Published:** 1861-03

**Authors:** 


					﻿Published Monthly, at $3 per Annum in Advance.
r
€
x
l.
&
a
&
Li
&
>*
z
&
z
x
5*
x
i
x.
I
I
L.
ft
X
X
ft
O
*
ft
1ft
Z
>
ft
O
>»
i
ft
Z
ft
c
IO
i
■H
Z
■<
ATLANTA
‘ ' A *•
’./JOURNAL.
J. G. WESTMORELAND, M. D.,
Professor of Materia Medica asd Medical Jcrisprudencb in the Atlanta Medical Coi.lecb,
EDITOR AND PROPRIETOR.
Pax et scientia, sed veritas sine timore. _
Vol. VI.	MARCH, 1861.	No. VII.
ATLANTA, GA:
ATLANTA INTELLIGKNCER PRI2NTT.
1801.
Each Number contains Sixty-Four Pages.
J
x
s
X
ft
ft
X
X
ft
ft
ft
£
ft
X
ft
&
©
ft
©
’<
>
5
X
i
j;
K
X
©
ft
ft
*■
ft
ft
X
X
*1
X
ft
ft
ft
ft
©
a
ft
ft
X
				

